# Rewiring carbon flow in *Synechocystis* PCC 6803 for a high rate of CO_2_-to-ethanol under an atmospheric environment

**DOI:** 10.3389/fmicb.2023.1211004

**Published:** 2023-05-31

**Authors:** E-Bin Gao, Junhua Wu, Penglin Ye, Haiyan Qiu, Huayou Chen, Zhen Fang

**Affiliations:** ^1^School of Life Sciences, Jiangsu University, Zhenjiang, Jiangsu, China; ^2^School of Environment and Safety Engineering, Jiangsu University, Zhenjiang, Jiangsu, China; ^3^Ningbo Women and Children's Hospital, Ningbo, China

**Keywords:** cyanobacteria, metabolic engineering, cofactor regeneration, CO_2_ fixation, photosynthetic cell factory

## Abstract

Cyanobacteria are an excellent microbial photosynthetic platform for sustainable carbon dioxide fixation. One bottleneck to limit its application is that the natural carbon flow pathway almost transfers CO_2_ to glycogen/biomass other than designed biofuels such as ethanol. Here, we used engineered *Synechocystis* sp. PCC 6803 to explore CO_2_-to-ethanol potential under atmospheric environment. First, we investigated the effects of two heterologous genes (pyruvate decarboxylase and alcohol dehydrogenase) on ethanol biosynthesis and optimized their promoter. Furthermore, the main carbon flow of the ethanol pathway was strengthened by blocking glycogen storage and pyruvate-to-phosphoenolpyruvate backflow. To recycle carbon atoms that escaped from the tricarboxylic acid cycle, malate was artificially guided back into pyruvate, which also created NADPH balance and promoted acetaldehyde conversion into ethanol. Impressively, we achieved high-rate ethanol production (248 mg/L/day at early 4 days) by fixing atmospheric CO_2_. Thus, this study exhibits the proof-of-concept that rewiring carbon flow strategies could provide an efficient cyanobacterial platform for sustainable biofuel production from atmospheric CO_2_.

## Graphical Abstract

Carbon flux rewiring enables high rate of CO_2_-to-ethanol in photosynthetic microorganism. 
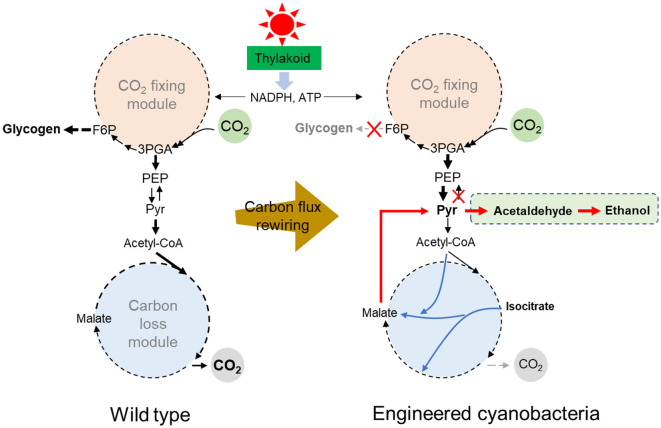

## 1. Introduction

The increased level of atmospheric greenhouse gas arises the concern of seeking environmentally friendly technologies to fix and even reuse CO_2_ as an energy chemical (Fang et al., 2021). Microbial CO_2_ fixation has received much attention because of its highly renewable reaction under mild conductions (Gassler et al., 2020; Satanowski and Bar-Even, 2020; Chen et al., 2023a,b). Among those biotechnologies, photo-driven CO_2_ bioconversion represents one of the sustainable strategies to generate carbon-neutral biofuels, such as ethanol and butanol (Liu et al., 2019; Velmurugan and Incharoensakdi, 2020; Fang et al., 2022). Thus, it is urgent to develop a photo-driven biosynthesis platform for CO_2_-to-biofuel production.

Cyanobacterium owns high photosynthesis efficiency (theoretical maximum is 8–10%) and has the potential to convert CO_2_ into biofuels through the Calvin–Benson–Bassham (CBB) cycle (Santos-Merino et al., 2021). Notably, it has successfully engineered cyanobacteria to assimilate CO_2_ and produce value-added chemicals, such as ethylene (Li et al., 2021), isoprene (Lindberg et al., 2010), ethanol (Gao et al., 2012), isobutanol (Miao et al., 2017), acetone (Lee et al., 2020), and *p*-coumaric acid (Gao et al., 2021). Ethanol as a simple but major renewable biofuel can be easily produced by introducing two heterologous enzymes (pyruvate decarboxylase and alcohol dehydrogenase) in cyanobacteria (Gao et al., 2012). The model cyanobacterium of *Synechocystis* sp. PCC 6803 (hereafter *Synechocystis*) shows double ethanol yield compared to other cyanobacteria such as *Synechococcus elongatus* PCC 7942 (Dexter and Fu, 2009). Furthermore, *Synechocystis* owns clear genetic background to assemble and engineer heterologous pathways, indicating the promising future of CO_2_-to-ethanol production (Zhang and Bryant, 2011).

Recently, many efforts have been explored to promote ethanol production in *Synechocystis*. Optimization of abiotic and biotic factors showed positive effects on cell growth and ethanol synthesis (Heidorn et al., 2011; Gao et al., 2012). Overexpressing the ethanol-producing steps or blocking the production of storage polymers (glycogen and polyhydroxybutyrate) was able to increase ethanol production (Namakoshi et al., 2016; Velmurugan and Incharoensakdi, 2020). The enhancement of carbon fixation in the CBB cycle also significantly improved the ethanol yield as well as cell growth (Liang et al., 2018; Roussou et al., 2021). In addition, co-culture engineering and modular engineering were systematic strategies to achieve high-level ethanol production in photosynthetic microorganisms (Liu et al., 2019; Velmurugan and Incharoensakdi, 2020). However, it is difficult to channel the fixed carbon atoms into the target product because of the imbalance of cell growth rate and ethanol byproduct accumulation (Luan et al., 2020). The above strategies are still challenging to adjust the ethanol pathway in one system and are rarely explored in systematic investigations on promoter optimization, byproduct blocking, and cofactor regeneration on ethanol accumulation. In addition, efficiently fixing atmospheric CO_2_ into ethanol via *Synechocystis* is still due to a lack of study.

Herein, to investigate the ethanol-producing potential of optimizing metabolic pathways, the engineered *Synechocystis* cells were genetically modified in a stepwise approach via inhibiting the phosphoenolpyruvate pathway from pyruvate, removing glycogen storage, and shunting carbon metabolic flux of the tricarboxylic acid cycle. This approach leads to proof-of-concept with high-efficient ethanol production directly from solar energy and atmospheric CO_2_ and significantly contributes to the sustainability of CO_2_-to-biofuel conversion.

## 2. Materials and methods

### 2.1. Strains and growth conditions

*Escherichia coli* DH5α carrying various plasmids were grown in LB medium, which contained special antibiotics such as 50 μg/ml spectinomycin (Sp^R^), 50 μg/ml kanamycin (Km^R^), or 25 μg/ml chloramphenicol (Cm^R^). *Synechocystis* cells were grown in the BG11 medium and cultured at light conductions (50 μmol photons m^−2^ s^−1^ and 30°C). Unless otherwise noted, appropriate antibiotics were added to the BG11 medium.

### 2.2. Plasmid construction for gene knockout

The pMD18-T vector (TaKaRa, Dalian) is used as a backbone to construct cyanobacterial plasmids, which are presented in Supplementary Table S1. Using PCR to amplify the fragments, the fragment and the vector were double-digested by recombinase (NEW ENGLAND BioLabs Beijing, China). Corresponding primers (SupplementaryTable S2) were used to clone up/down-fragments of the *Synechocystis* genome, and T4 ligase (NEB, Beijing) was used for ligation. The recombinant pBE406 plasmid (containing 600 bp upstream/downstream *slr0168* and spectinomycin resistance gene) for gene knockout is shown in Supplementary Figure S2. Similarly, pMD-slr0301-Ω and pMD-slr1176-Ω were constructed. Otherwise, to construct a recombinant ethanol pathway, the synthesized *pdc* and *yqhD* genes (Sangon Biotech Co Ltd., Shanghai) coupled with promoters (*PpetE* or *PpsbA2s*) and *TrbcL* terminator were designed in pBE02/pBE03 (see the target genes including other resistance genes in Supplementary Table S1). Plasmid pBE09 was constructed by inserting the *PpsbA2s-maeB* expression cassette (*maeB* gene cloned from *E. coli*) and *TrbcL* terminator into the pMD-slr1176-Ω vector.

### 2.3. Engineered cyanobacteria construction

*Synechocystis* cells collected at the exponential phase (~1 OD_730_) were washed with a fresh BG11 medium three times, and then mixed with plasmids (100 ng DNA to 100 μl cyanobacteria) for 5 h and illuminated incubation at 30°C. The above mixture was streaked on a sterile filter membrane for another 24 h of illuminated incubation on the BG11 solid medium. To select the corrected mutant, the filter membrane was further transferred to a solid BG11 medium with corresponding antibiotics. After 2 weeks, single clones sub-cultured on solid plates were isolated in a liquid BG11 medium for analysis. All the strains referred to in this study are presented in Table 1.

**Table 1:** Engineered strains constructed in this experiment.

**Mutant strains**	**Gene cluster**	**Productivity**
SYN001	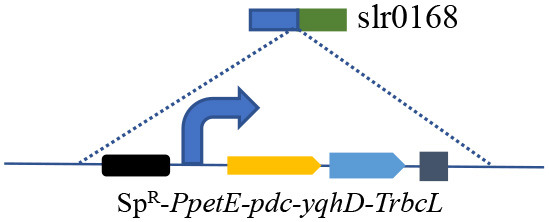	33 mg/L/day
SYN002	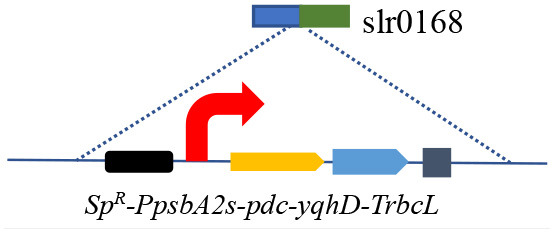	68 mg/L/day
SYN003	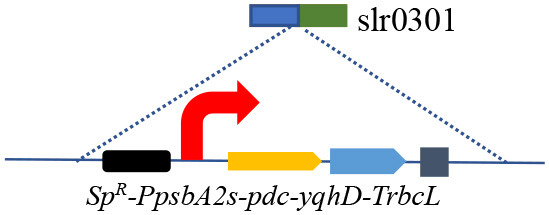	85 mg/L/day
SYN007	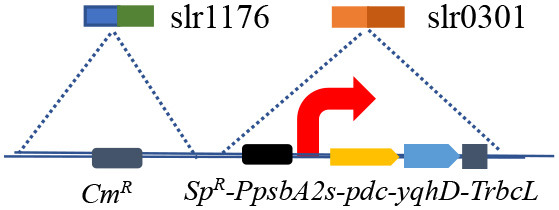	98 mg/L/day
SYN009	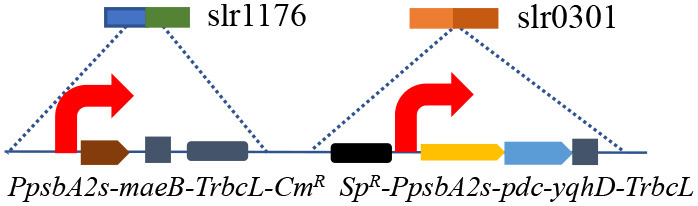	248 mg/L/day

The *slr0301* gene is a gene encoding phosphoenolpyruvate synthase (PpsA) in the genome of *Synechocystis* sp. PCC6803, which catalyzes the conversion of pyruvate to phosphoenolpyruvate. The absence of this gene increases the accumulation of the intermediate pyruvate. The *slr1176* gene is a gene encoding glucose-1-phosphate adenylate transferase in the genome of PCC6803, which catalyzes the conversion of glucose-1-phosphate (G1P) to ADP-glucose. It is a major rate-limiting enzyme in glycogen synthesis, and its absence can lead to complete inhibition of glycogen synthesis. *The slr0168* is a neutral gene in PCC6803 algae cells, and knocking out this gene has no effect on the growth of algae cells, making it a commonly used expression platform.

### 2.4. Ethanol production and analytical methods

For ethanol production, all the mutants were cultured in a fresh BG11 medium with an initial 0.1 OD_730_ and cultivated photoautotrophically in a flask (50 μmol photons m^−2^ s^−1^ without additional CO_2_ injection). Notably, the BG11 medium of SYN001 contains 500 nM copper ions to induce the expression of ethanol-producing genes (Ghassemian et al., 1994; Choi and Park, 2016). After centrifugation and filtration, supernatant with ethanol was submitted for high-performance liquid chromatography (HPLC) analysis using an Aminex HPX-87H column (Bio-Rad, United States) (Seo et al., 2017).

### 2.5. Transcription level analysis

*Synechocystis* wild-type culture and mutants at 0.6 OD_730_ were collected after centrifugation (3,500 × *g*, 15 min, 4°C). RNA extraction and quantitative reverse transcription PCR (RT-qPCR) analysis were performed according to the previous methods (Gao et al., 2012). The relative transcription levels of targeted genes were estimated using the calculation method of 2^−ΔΔCT^, in which a higher ΔCT value means low transcription (Livak and Schmittgen, 2001). The endogenous 16S rRNA was set as a reference gene. All experimental groups were carried out with three biological replicates.

### 2.6. Statistical analysis

All statistical analyses were performed using GraphPad Prism (version 8.01, United States). The difference in this study was compared by unpaired *t*-test and statistical significance was set at *p* < 0.05. ^**^ represents *p* < 0.01 and ^***^ represents *p* < 0.001.

## 3. Results and discussion

### 3.1. Synthetic ethanol pathway optimization

Generally, there are two precursors (pyruvate and acetyl-CoA) that are involved in ethanol synthesis (Gao et al., 2012). To determine the optimal ethanol pathway in engineered *Synechocystis*, the concentrations of those two metabolites were investigated (Supplementary Figure S1). Interestingly, pyruvate linking to the CBB cycle and tricarboxylic acid (TCA) cycle exhibited higher concentration (1.05 μmol/gDW, approximately three times that of acetyl-CoA), indicating that pyruvate was more suitable to serve as the ethanol precursor. Therefore, we attempted to optimize the carbon flow network by selecting a strong pyruvate-acetaldehyde-ethanol pathway.

**Figure 1:**
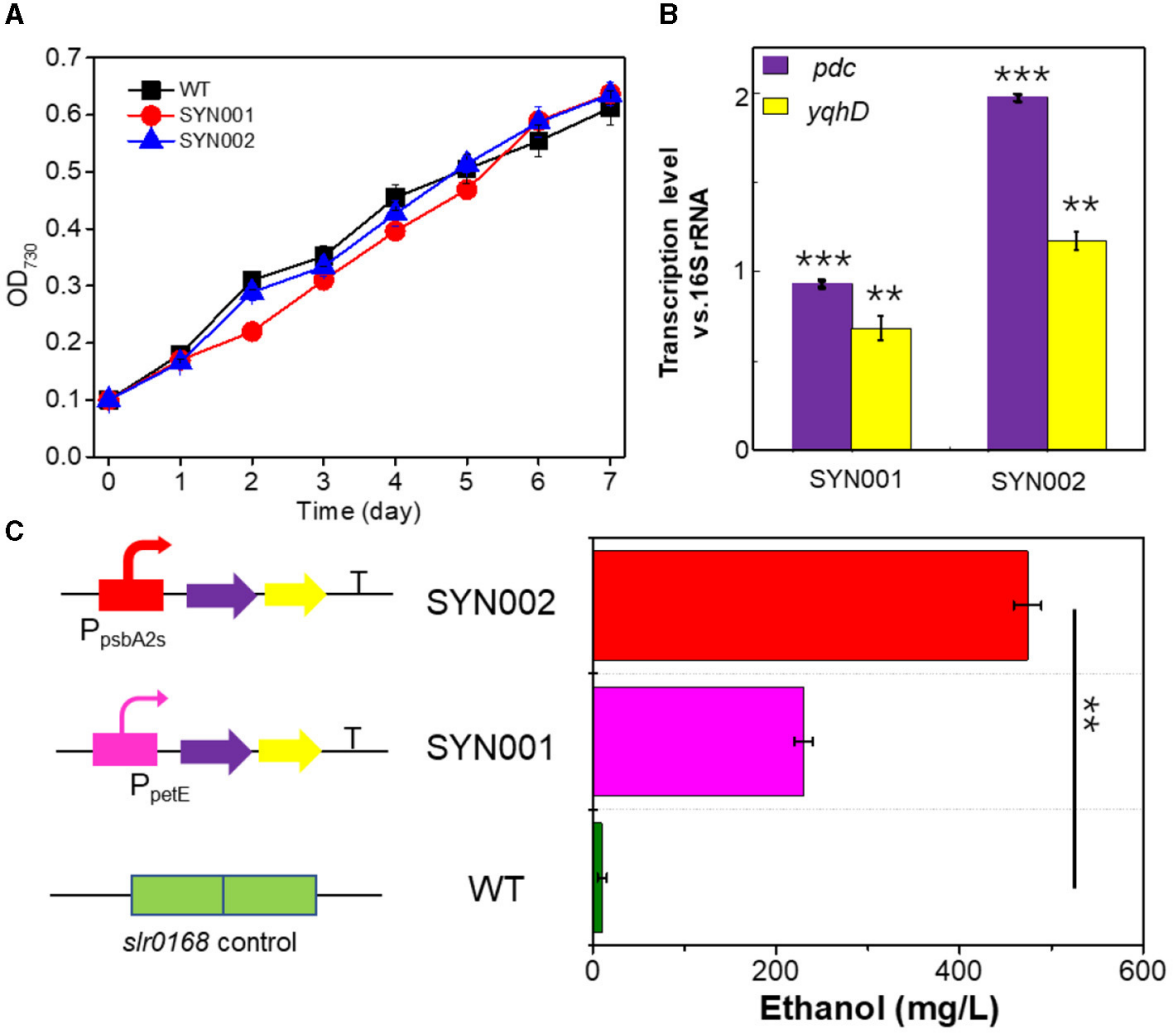
Construction of synthetic ethanol pathway in cyanobacteria. **(A)** Cell growth curves of wild-type and recombinant cyanobacteria. **(B)** Transcription levels of *pbd-yqhd* in various *Synechocystis* recombinants. **(C)** Ethanol yields under the control of various promoters. An unpaired *t*-test (^**^p < 0.01, ^***^p < 0.001) was used.

The synthetic ethanol pathway contained pyruvate decarboxylase (PDC) from *Zymomonas mobilis* and alcohol dehydrogenase (YqhD) from *Escherichia coli* (Supplementary Figure S2). Those two enzymes exhibited high activities to convert pyruvate into acetaldehyde and subsequently reduce acetaldehyde into ethanol in other microorganisms (Atsumi et al., 2009; Gao et al., 2012). The *slr0168* gene not affecting cell growth or photosynthesis was primarily chosen as an exchange site according to previous reports (Dexter and Fu, 2009; Gao et al., 2012). Vectors containing upstream/downstream *slr0168* gene, antibiotic resistance gene (spectinomycin, Sp^R^), various promoters, target genes (*pdc*-*yqhD*), and terminator *TrbcL* were constructed and integrated into the *Synechocystis* genome to obtain stable ethanol-producing recombinants (Supplementary Figure S3). Those two strains (SYN001 and SYN002) showed similar growth rates to wild-type *Synechocystis* (Figure 1A).

Subsequently, promoter optimization to adjust the transcription level of *pdc* and *yqhD* was conducted. The RT-qPCR results showed that strong promoter *P*_*psbA*2*s*_ in recombinant SYN002 obviously improved *pdc*-*yqhD* transcription levels, ~2-fold compared to medium-level promoter *P*_*petE*_ in SYN001 (Figure 1B and Supplementary Figure S4). As expected, both recombinants achieved obvious accumulation of ethanol (rarely detected in wild-type *Synechocystis*), and SYN002 yielded the highest titer (474 mg/L) at 7 days (Figure 1C). Thus, a basic cyanobacterium with photosynthetic CO_2_-to-ethanol ability was obtained.

### 3.2. Effect of phosphoenolpyruvate synthase and glycogen synthesis knockout

**Figure 2:**
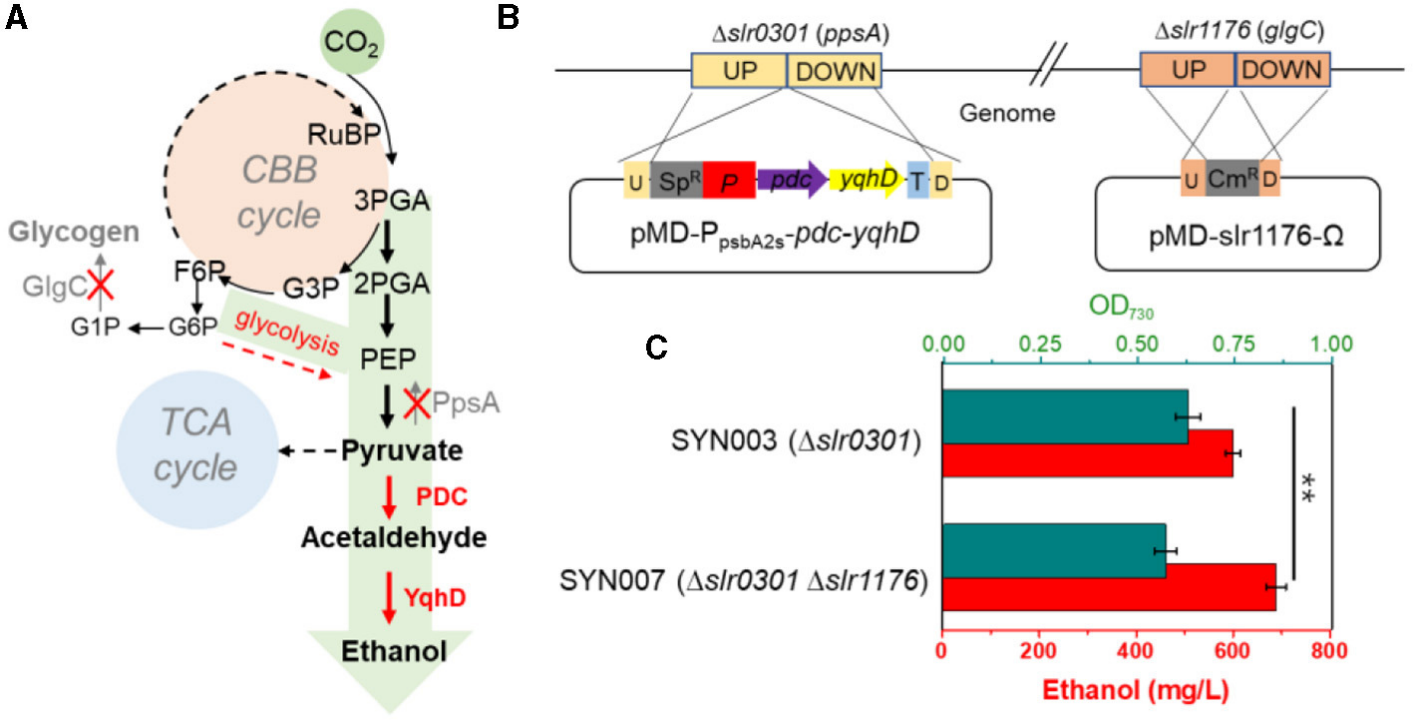
Effects of blocking carbon loss on CO_2_-to-ethanol production. **(A)** Knockout sites on the pathway map. The gray font words of GlgC and PpsA are glycogen synthase and phosphoenolpyruvate synthase, respectively. **(B)** Heterologous genes exchange and cyanobacteria gene knockout schematics. **(C)** Cell density and ethanol yield of various *Synechocystis* recombinants under photosynthetic process. An unpaired *t*-test (^**^p < 0.01, ^***^p < 0.001) was used.

Blocking carbon loss (phosphoenolpyruvate backflow and glycogen synthesis) was conducted to learn their effects on CO_2_-to-ethanol production (Figure 2A). From metabolic network analysis (Supplementary Figures S1, S2) and literature investigation (Angermayr et al., 2014; Dienst et al., 2014), we learned that native *Synechocystis* could remarkably turn pyruvate back into the upstream module via highly active phosphoenolpyruvate synthase (PpsA, referred to *slr0301* gene). To abolish competitive consumption of pyruvate, we constructed a new cassette (pMD-P_psbA2s_-*pdc-yqhD*) to exchange the *slr0301* gene on the genome (Figure 2B). The newly obtained recombinant SYN003 (Δ*slr0301*) exhibited 600 mg/L ethanol yield after 7 days of photosynthetic CO_2_ conversion (Figure 2C) and approximately 1.3-fold improvement compared to PpsA-existed SYN002. Furthermore, to enhance photosynthetic carbon flux toward the CO_2_-to-ethanol pathway, a key gene *slr1176* related to glycogen synthesis was knocked out via gene exchange cassette (pMD-*slr1176*-Ω) (Figure 2B). Impressively, the ethanol yield was further improved via double-knockout recombinant SYN007 (Δ*slr0301* Δ*slr1176*) and obtained more than 700 mg/L titer (Figure 2C). Pyruvate was deduced as a carbon sink in the Embden–Meyerhof–Parnas pathway according to the previous study of glycogen synthesis abolishment (Van Der Woude et al., 2014). The increased carbon flux of CO_2_-to-pyruvate probably supported the pyruvate-utilizing reaction of ethanol accumulation. Interestingly, it slightly inhibited cell growth when blocking glycogen synthesis at the *slr1176* site (Figure 2C). We deduced that the shift of excessive carbon from the glycolytic pathway and pentose phosphate pathway to ethanol pathway resulted in carbon deficiency of biomass synthesis (Young et al., 2011).

### 3.3. Effect of malic enzyme overexpression on ethanol production

**Figure 3:**
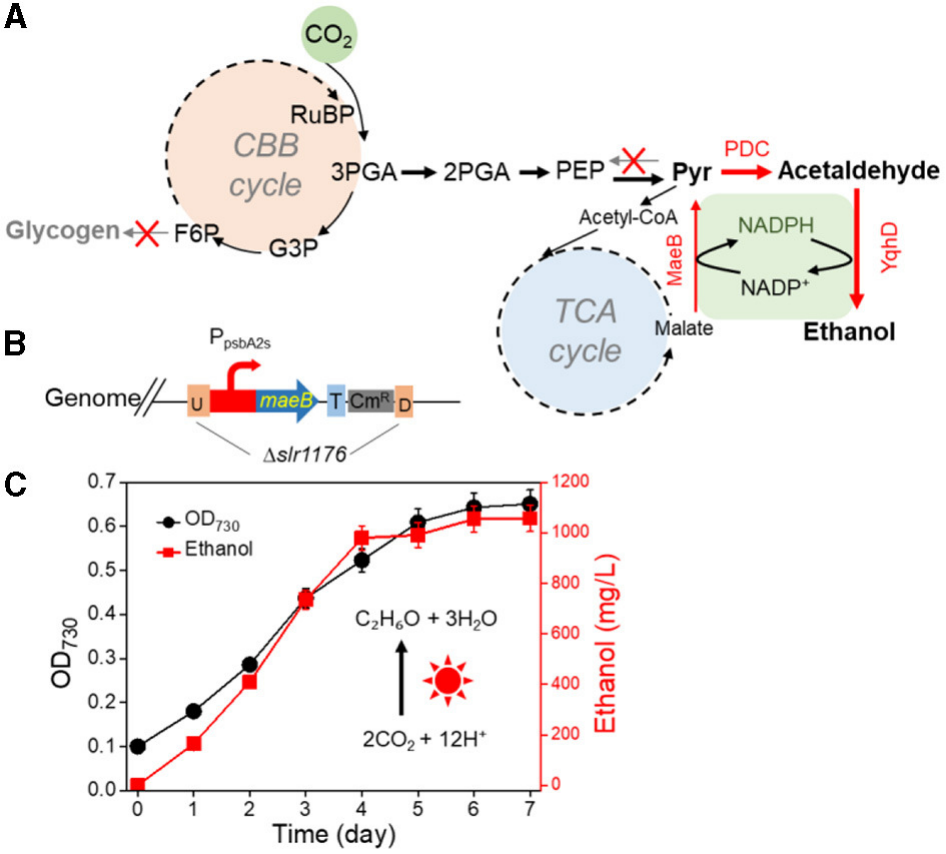
Effects of malic enzyme overexpression on ethanol production. **(A)** Pathway construction schematics. **(B)** The gene exchange of cassette of new recombinant. **(C)** The cell growth and photosynthetic ethanol production time curve using atmospheric CO_2_.

The engineered strains in cell proliferation stage should use the TCA cycle to support biomass synthesis, however, resulting in carbon atom loss (Zhang and Bryant, 2011). Thus, we designed a simple pathway to modify the TCA cycle by improving glyoxylate flux to reduce the carbon loss between isocitrate and succinate (Supplementary Figure S2). Malate close to the end of the TCA cycle was selected as the key metabolite for carbon recycling through an NADP^+^-dependent malic enzyme from *E. coli* (Yoshikawa et al., 2015), which not only converted malate into pyruvate but also increased pyruvate and NADPH pool (Figure 3A). The *maeB* gene was introduced into the *slr1176* site of SYN003, establishing the recombinant SYN009 (Figure 3B). Intriguingly, the cell growth of *Synechocystis* was rescued, and the SYN009 showed a little fast proliferation after 3 days of photoautotrophic growth (Supplementary Figure S6). Under simulated sunlight source, SYN009 used CO_2_ as a sole carbon source to produce 1.09 g/L ethanol (Figure 3C and Supplementary Figure S5). Impressively, the time curve showed that before 4 days, SYN009 achieved 248 mg/L/day productivity, the fastest accumulation rate compared to other literature studies (Table 2).

**Table 2:** Baseline sociodemographic characteristics of participants in the study.

**Year**	**Cyanobacterial type**	**Engineering strategy**	**Yield (mg/L)**	**Time (day)**	**Productivity (mg/L/day)**	**Ref**.
1999	*Synechococcus* PCC7942	Overexpress *pdc* and *adh* from Z*ymomonas mobilis*	230	7	33	Deng and Coleman (1999)
2009	*Synechocystis* PCC6803	Light driven *psbAII* promoter, *pdc*, and *adh* overexpression	460	6	77	Dexter and Fu (2009)
2012	*Synechocystis* PCC6803	Introduce *Z. mobilis* PDC, disrupt poly-β-hydroxybutyrate biosynthesis, ADH, and culturing conditions optimization	5,500	26	212	Gao et al. (2012)
2014	*Synechocystis* PCC6803	P*petJ*-*pdc-adhII* (*Z. mobilis*) at *slr1192*	432	18	24	Dienst et al. (2014)
2016	*Synechocystis* PCC6803	Excessive NADPH production by *zwf* over-expression	590	14	42	Choi and Park (2016)
2016	*Synechocystis* PCC6803	Combinatorial deletions of *glgC* and *phaCE*, high cell density culture	~3,000	3	1,080–2,010^a^	Namakoshi et al. (2016)
2018	*Synechocystis* PCC6803	Four CBB cycle enzymes (RuBisCO, FBP/SBPase, TK, FBA) were co-overexpressed with PDC and ADH	700	7	100	Liang et al. (2018)
2020	*Synechococcus* PCC7002	Remove glycogen synthesis genes and introduce ethanologenic cassettes	2,200	10	220	Wang et al. (2020)
2020	*Synechocystis* PCC6803	Co-cultivation of two engineered strains*: pdc*-*adh* overexpression and *glgC*-*phaA* knockout	4,500	20	225	Velmurugan and Incharoensakdi (2020)
2021	*Synechococcus* PCC7942	Co-expression of *ictB, ecaA*, and *groESL*	200	2	100	Chou et al. (2021)
2021	*Synechocystis* PCC6803	Overexpress two enzymes (FBA + TK, FBP/SBPase + FBA) of CBB cycle	1,200	20	60	Roussou et al. (2021)
2023	*Synechocystis* PCC6803	Remodel carbon flow (integrate *pdc-yqhD*, knockout *glgC-ppsA*, overexpress *maeB*)	992	4	248	This study

a Using high cell density (OD_730_ = 50) at an initial time and its productivities were determined at first 24 h.

The metabolic flux imbalance between metabolism and synthesis is a big challenge limiting target product yield in microbial cell factories (Oliver et al., 2013). Choosing suitable promoters to overexpress PDC/YqhD has been usually considered to enhance the carbon flux toward ethanol (Dexter and Fu, 2009; Gao et al., 2012), which also exhibited positive results of ethanol production in this study (Supplementary Figure S5). In addition, the overexpression of key enzymes in the CBB cycle was another important strategy to supply sufficient carbon flux in the form of 3-phosphoglycerate (Liang et al., 2018; Roussou et al., 2021). Compared to previous reports (Table 2), the carbon flow optimization strategy in our study consumed the minimum number of days to achieve the highest ethanol productivity of ~248 mg/L/day (Table 1). It indicated that a comprehensive and precise adjustment of carbon flow is promising to improve CO_2_-to-ethanol production in cyanobacteria. Notably, this photosynthetic cell factory still faces the challenge of cell density (Supplementary Figure S6), such as only ~0.6 OD_730_ increase after 7 days of cultivation under light and atmospheric CO_2_. New strategies, such as co-cultivation and batch culture with high density, can probably yield outstanding ethanol production (Namakoshi et al., 2016; Velmurugan and Incharoensakdi, 2020).

## 4. Conclusion

We developed a cyanobacterial platform that was entitled to convert atmospheric CO_2_ into ethanol at high efficiency via stepwise optimization of carbon flow. It showed that carbon flow rewiring strategies, such as integrating strong pyruvate-acetaldehyde-ethanol pathway, blocking carbon loss via inhibition of PEP synthase activity and glycogen synthesis, and recycling carbon atoms via overexpression of exogenous malic enzyme, were beneficial to ethanol synthesis. This study provides a proof-of-concept to create a photosynthetic cell factory that could be further remodeled and optimized for higher CO_2_-to-biofuel production.

## Data availability statement

The original contributions presented in the study are included in the article/Supplementary material, further inquiries can be directed to the corresponding authors.

## Author contributions

E-BG conceived the original idea, carried out the experiment, and wrote the manuscript with input from all authors. ZF and HQ interpreted the results contributed to the final version of the manuscript. JW and HC aided in interpreting the results and worked on the manuscript. ZF and PY contributed to the analysis of the results, the writing of the manuscript, and supervised the project. All authors provided critical feedback and helped shape the research, analysis, and manuscript.
