# Supplementary material for: Rewiring carbon flow in Synechocystis PCC 6803 for a high rate of CO2-to-ethanol under an atmospheric environment
Source: Front Microbiol. 2023 May 31;14:1211004. doi: 10.3389/fmicb.2023.1211004 (PMC10265512; doi:10.3389/fmicb.2023.1211004)
Supplement: Supplementary file 1 [file Data_Sheet_1.docx]

*Supporting Information*


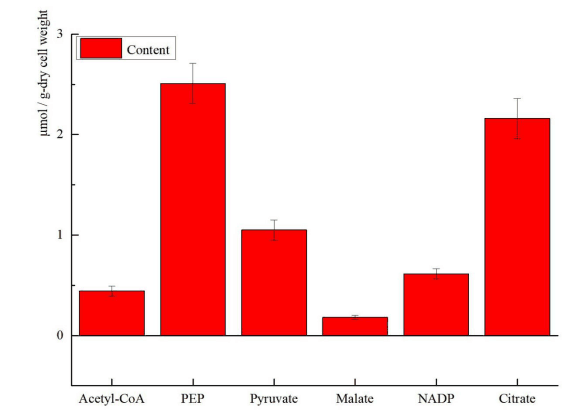


**Fig. S1** Intracellular concentration of main metabolites.


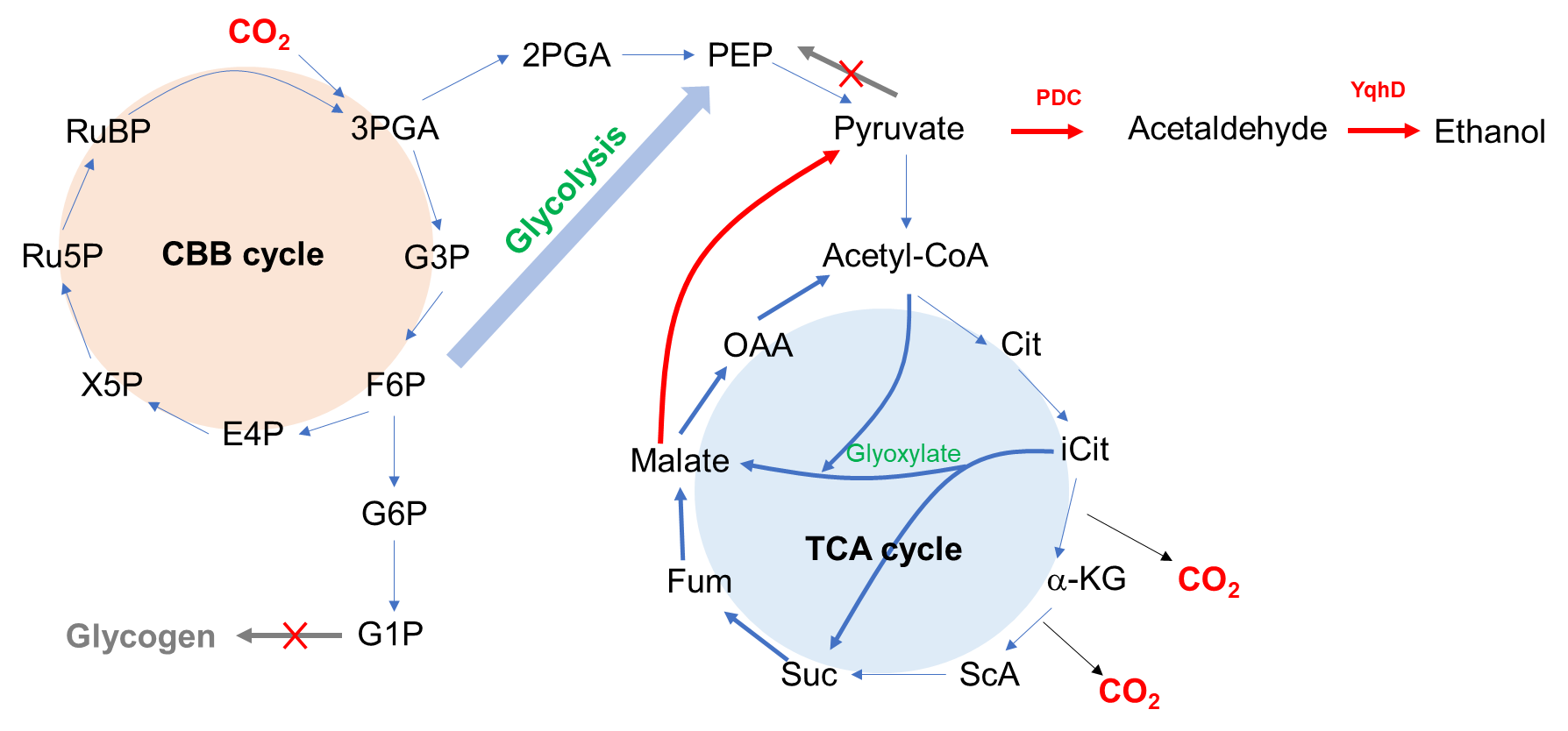


**Fig. S2** Overview of metabolic engineering pathway of *Synechocystis* in this study. **RuBP**, ribulose-1,5-bisphosphate; **G1P**, glucose-1-phosphate; **G6P**, glucose-6-phosphate; **2PGA**, 2-phosphoglyceric acid; **3PGA**, 3-phosphoglycerate; **F6P**, fructose-6-phosphate; **PEP**, phosphoenolpyruvate; **Pyr**, Pyruvate. Black arrows indicate native carbon flow, red arrows indicate newly created pathway, and light blue arrows indicate probably reinforced pathway. Knockout pathways are red crosses.


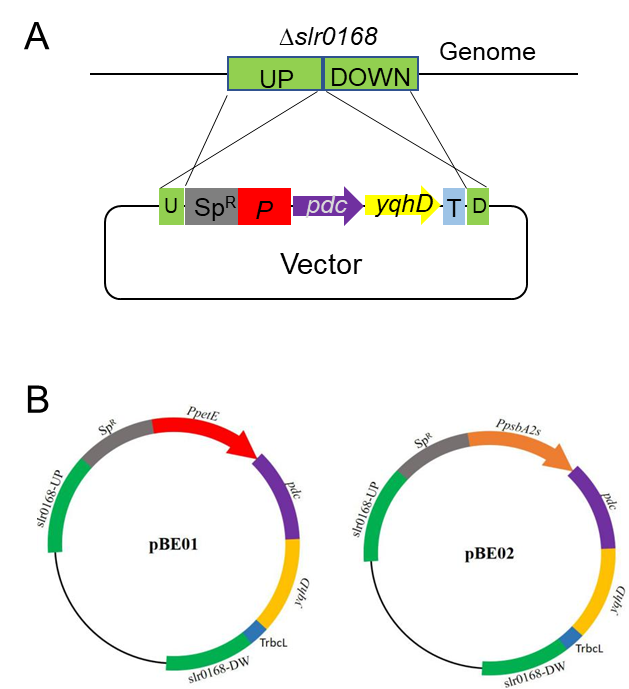


**Fig. S3** (A) Vector with *pbd-yqhd* cassette to exchange *slr0168* site on *Synechocystis* genome. (B) Vectors maps of cassette for gene exchange in *Synechocystis.* U/D are homology arms of *slr0168*, P is promoter, and T is terminator.


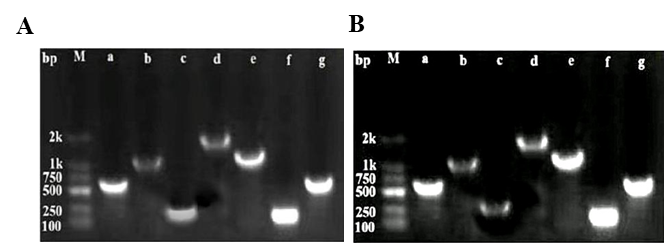


**Fig. S4** PCR analysis of the insertion of ethanol biosynthetic pathway in the engineered strain SYN001 (**A**) and SYN002 (**B**), respectively. M-DNA marker, a-upstream of the slr0168 gene, b-Spectinomycin resistance gene, c-promoter *PpetE* (lift) or *PpbsA2s* (right), d-*pdc* gene, e-*yqhD* gene, f-*Synechocystis* TrbcL terminator, g-downstream of the slr0168 gene.

**Ethanol content (mg/L)**

B

Ethanol

SYN007

Wild-type

SYN009

SYN003

SYN002

SYN001

Water

A

**Retention time (min)**

**LC counts (mAμ)**

A

LC counts (mAu)

Time (min)

Water

| **Stain No.** | **SYN001** | **SYN002** | **SYN003** | **SYN007** | **SYN009** |
| --- | --- | --- | --- | --- | --- |
| ***pdc*** | **+** | **+** | **+** | **+** | **+** |
| ***yqhD*** | **+** | **+** | **+** | **+** | **+** |
| ***PpetE*** | **+** | **--** | **--** | **--** | **--** |
| ***PpsbA2s*** | **--** | **+** | **+** | **+** | **+** |
| ***slr0301*** | **+** | **+** | **--** | **--** | **--** |
| ***slr1176*** | **+** | **+** | **+** | **--** | **--** |
| ***maeB*** | **--** | **--** | **--** | **--** | **+** |

**Fig. S5.** The detection and production of ethanol in the engineered *Synechocystis* strains. **A**, HPLC counts versus retention time is shown for the detection of ethanol in different strains. **B**, Ethanol production in different engineered strains. Error bars represent standard deviations of three biological replicates.

**Time (days)**

**OD_730_**

**Fig. S6.** The growth of *Synechocystis* sp. Wild-type (magenta), SYN003 (black), SYN004 (dark yellow), SYN007 (blue) under the autotrophic conditions. Each data point represents the mean and standard deviation of three independent experiments.

**Table S1**. Gene sequences in this study.

| **Name** | **Sequence** |
| --- | --- |
| *pdc* | ATGAGTTATACCGTGGGCACCTATTTGGCCGAACGCTTGGTGCAAATTGGCTTGAAACATCATTTTGCCGTGGCCGGCGATTATAACTTAGTGTTGTTAGATAATTTATTGTTGAATAAAAATATGGAACAAGTGTATTGTTGTAATGAATTGAATTGTGGCTTTAGTGCCGAAGGCTATGCCCGCGCCAAAGGCGCCGCCGCCGCCGTGGTGACCTATTCCGTGGGCGCCTTGTCCGCCTTTGATGCCATTGGCGGTGCTTATGCCGAAAATTTGCCCGTGATTTTGATTAGTGGCGCCCCCAATAATAATGATCATGCCGCCGGCCATGTGTTGCATCATGCCTTGGGCAAAACCGATTATCACTATCAATTAGAAATGGCCAAAAATATTACCGCCGCCGCCGAAGCCATTTATACCCCCGAAGAAGCCCCCGCCAAAATTGATCATGTGATTAAAACCGCCTTGCGCGAGAAAAAACCCGTGTATTTGGAAATTGCCTGTAATATTGCCTCCATGCCCTGTGCCGCCCCCGGCCCCGCCTCCGCCTTGTTTAATGATGAAGCCTCCGATGAAGCCAGTCTGAATGCCGCCGTGGAAGAAACCTTAAAATTTATTGCCAATCGCGATAAAGTGGCCGTGTTGGTGGGCTCCAAATTGCGGGCCGCGGGTGCCGAAGAAGCCGCCGTGAAATTTGCCGATGCCTTAGGCGGCGCCGTGGCCACCATGGCCGCCGCCAAATCCTTCTTTCCCGAAGAAAATCCCCATTATATTGGGACCTCCTGGGGTGAAGTGAGTTATCCCGGCGTGGAAAAAACCATGAAAGAAGCCGATGCCGTGATTGCCTTAGCCCCCGTGTTTAATGATTATTCCACCACCGGCTGGACTGATATTCCCGATCCCAAAAAATTAGTGTTAGCCGAACCCCGGTCCGTGGTGGTGAATGGCATTCGGTTTCCCAGTGTGCATTTGAAAGATTATTTGACCCGGTTGGCCCAAAAAGTTAGTAAGAAAACCGGCGCCTTGGATTTCTTTAAAAGTTTGAATGCCGGCGAATTGAAAAAAGCCGCCCCCGCCGATCCCAGCGCTCCCTTGGTGAATGCCGAAATTGCCCGGCAAGTGGAAGCCTTGCTGACCCCCAATACCACCGTGATTGCCGAAACCGGTGATTCCTGGTTTAATGCCCAACGCATGAAATTGCCCAATGGCGCCCGCGTGGAATATGAAATGCAGTGGGGGCATATCGGTTGGTCCGTGCCCGCCGCTTTTGGATATGCCGTGGGTGCCCCCGAACGCCGCAATATTTTAATGGTGGGCGATGGGTCCTTTCAATTAACCGCCCAAGAAGTGGCCCAAATGGTGCGGTTAAAATTGCCCGTGATTATTTTCTTGATTAATAATTATGGTTATACCATTGAAGTGATGATTCATGATGGGCCCTATAATAATATTAAAAATTGGGATTATGCCGGCTTGATGGAAGTGTTTAATGGTAATGGCGGCTATGATTCCGGCGCCGGTAAAGGCTTGAAAGCCAAAACCGGCGGCGAATTAGCCGAAGCCATTAAAGTGGCCTTGGCCAATACCGATGGCCCCACCTTAATTGAATGTTTTATTGGCCGGGAAGATTGCACCGAAGAATTGGTGAAATGGGGTAA |
| *yqhD* | ATGAACAACTTTAATCTGCACACCCCAACCCGCATTCTGTTTGGTAAAGGCGCAATCGCTGGTTTACGCGAACAAATTCCTCACGATGCTCGCGTATTGATTACCTACGGCGGCGGCAGCGTGAAAAAAACCGGCGTTCTCGATCAAGTTCTGGATGCCCTGAAAGGCATGGACGTGCTGGAATTTGGCGGTATTGAGCCAAACCCGGCTTATGAAACGCTGATGAACGCCGTGAAACTGGTTCGCGAACAGAAAGTGACTTTCCTGCTGGCGGTTGGCGGCGGTTCTGTACTGGACGGCACCAAATTTATCGCCGCAGCGGCTAACTATCCGGAAAATATCGATCCGTGGCACATTCTGCAAACGGGCGGTAAAGAGATTAAAAGCGCCATCCCGATGGGCTGTGTGCTGACGCTGCCAGCAACCGGTTCAGAATCCAACGCAGGCGCGGTGATCTCCCGTAAAACCACAGGCGACAAGCAGGCGTTCCATTCTGCCCATGTTCAGCCGGTATTTGCCGTGCTCGATCCGGTTTATACCTACACCCTGCCGCCGCGTCAGGTGGCTAACGGCGTAGTGGACGCCTTTGTACACACCGTGGAACAGTATGTTACCAAACCGGTTGATGCCAAAATTCAGGACCGTTTCGCAGAAGGCATTTTGCTGACGCTAATCGAAGATGGTCCGAAAGCCCTGAAAGAGCCAGAAAACTACGATGTGCGCGCCAACGTCATGTGGGCGGCGACTCAGGCGCTGAACGGTTTGATTGGCGCTGGCGTACCGCAGGACTGGGCAACGCATATGCTGGGCCACGAACTGACTGCGATGCACGGTCTGGATCACGCGCAAACACTGGCTATCGTCCTGCCTGCACTGTGGAATGAAAAACGCGATACCAAGCGCGCTAAGCTGCTGCAATATGCTGAACGCGTCTGGAACATCACTGAAGGTTCCGATGATGAGCGTATTGACGCCGCGATTGCCGCAACCCGCAATTTCTTTGAGCAATTAGGCGTGCCGACCCACCTCTCCGACTACGGTCTGGACGGCAGCTCCATCCCGGCTTTGCTGAAAAAACTGGAAGAGCACGGCATGACCCAACTGGGCGAAAATCATGACATTACGTTGGATGTCAGCCGCCGTATATACGAAGCCGCCCGCTAA |
| Sp^R^ | CCTGTAGAGAAGAGTCCCTGAATATCAAAATGGTGGGATAAAAAGCTCAAAAAGGAAAGTAGGCTGTGGTTCCCTAGGCAACAGTCTTCCCTACCCCACTGGAAACTAAAAAAACGAGAAAAGTTCGCACCGAACATCAATTGCATAATTTTAGCCCTAAAACATAAGCTGAACGAAACTGGTTGTCTTCCCTTCCCAATCCAGGACAATCTGAGAATCCCCTGCAACATTACTTAACAAAAAAGCAGGAATAAAATTAACAAGATGTAACAGACATAAGTCCCATCACCGTTGTATAAAGTTAACTGTGGGATTGCAAAAGCATTCAAGCCTAGGCGCTGAGCTGTTTGAGCATCCCGGTGGCCCTTGTCGCTGCCTCCGTGTTTCTCCCTGGATTTATTTAGGTAATATCTCTCATAAATCCCCGGGTAGTTAACGAAAGTTAATGGAGATCAGTAACAATAACTCTAGGGTCATTACTTTGGACTCCCTCAGTTTATCCGGGGGAATTGTGTTTAAGAAAATCCCAACTCATAAAGTCAAGTAGGAGATTAATTC |
| Cm^R^ | TGATCGGCACGTAAGAGGTTCAACTTTCACCATAATGAAATAAGATCACTACCGGGCGTATTTTTTGAGTTATCGAGATTTTCAGGAGCTAAGGAAGCTAAAATGGAGAAAAAAATCACTGGATATACCACCGTTGATATATCCCAATGGCATCGTAAAGAACATTTTGAGGCATTTCAGTCAGTTGCTCAATGTACCTATAACCAGACCGTTCAGCTGGATATTACGGCCTTTTTAAAGACCGTAAAGAAAAATAAGCACAAGTTTTATCCGGCCTTTATTCACATTCTTGCCCGCCTGATGAATGCTCATCCGGAATTCCGTATGGCAATGAAAGACGGTGAGCTGGTGATATGGGATAGTGTTCACCCTTGTTACACCGTTTTCCATGAGCAAACTGAAACGTTTTCATCGCTCTGGAGTGAATACCACGACGATTTCCGGCAGTTTCTACACATATATTCGCAAGATGTGGCGTGTTACGGTGAAAACCTGGCCTATTTCCCTAAAGGGTTTATTGAGAATATGTTTTTCGTCTCAGCCAATCCCTGGGTGAGTTTCACCAGTTTTGATTTAAACGTGGCCAATATGGACAACTTCTTCGCCCCCGTTTTCACCATGGGCAAATATTATACGCAAGGCGACAAGGTGCTGATGCCGCTGGCGATTCAGGTTCATCATGCCGTTTGTGATGGCTTCCATGTCGGCAGAATGCTTAATGAATTACAACAGTACTGCGATGAGTGGCAGGGCGGGGCGTAACCAT |
| *TrbcL* | TACCGTTACAGTTTTGGCAATTACTAAAAAACTGACTTCAATTCAATGTTAGCCCGCTCCCGCGGGTTTTTTGTTGCTTTTTCACAGTGACTATAGGTAATCAGCAACACAATACGGCCCTGTTCTTTGGACAGTTTTTGTATAATGTTGACCGCATCCTGACCGGATTTTTTATCTAAGTGGGGAA |
| *P_petE_* | CAAGGATTCATAGCGGTTGCCCAATCTAACTCAGGGAGCGACTTCAGCCCACAAAAAACACCACTGGGCCTACTGGGCTATTCCCATTATCATCTACATTGAAGGGATAGCAAGCTAATTTTTATGACGGCGATCGCCAAAAACAAAGAAAATTCAGCAATTACCGTGGGTAGCAAAAAATCCCCATCTAAAGTTCAGTAAATATAGCTAGAACAACCAAGCATTTTCGGCAAAGTACTATTCAGATAGAACGAGAAATGAGCTTGTTCTATCCG**CCCGGG**GCTGAGGCTGTATAATCTACGACGGGCTGTCAAACATTGTGATACCATGGGCAGAAGAAAGGAAAAACGTCCCTGATCGCCTTTTTGGGCACGGAGTAGGGCGTTACCCCGGCCCGTTCAACCACAAGTCCCTATAGATACAATCGCCAAGAA |
| *P_psbA2s_* | TCACCATTTGGACAAAACATCAGGAATTCTAATTAGAAAGTCCAAAAATTGTAATTTAAAAAACAGTCAATGGAGAGCATTGCCATAAGTAAAGGCATCCCCTGCGTGATAAGATTACCTTCAGAAAACAGATAGTTGCTGGGTTATCGCAGATTTTTCTCGCAACCAAATAACTGTAAATAATAACTGTCTCTGGGGCGACGGTAGGCTTTATATTGCCAAATTTCGCCCGTGGGAGAAAGCTAGGCTATTCAATGTTTATGGAGGACTGACCTAG |
| *maeB* | ATGGATGACCAGTTAAAACAAAGTGCACTTGATTTCCATGAATTTCCAGTTCCAGGGAAAATCCAGGTTTCTCCAACCAAGCCTCTGGCAACACAGCGCGATCTGGCGCTGGCCTACTCACCAGGCGTTGCCGCACCTTGTCTTGAAATCGAAAAAGACCCGTTAAAAGCCTACAAATATACCGCCCGAGGTAACCTGGTGGCGGTGATCTCTAACGGTACGGCGGTGCTGGGGTTAGGCAACATTGGCGCGCTGGCAGGCAAACCGGTGATGGAAGGCAAGGGCGTTCTGTTTAAGAAATTCGCCGGGATTGATGTATTTGACATTGAAGTTGACGAACTCGACCCGGACAAATTTATTGAAGTTGTCGCCGCGCTCGAACCAACCTTCGGCGGCATCAACCTCGAAGACATTAAAGCGCCAGAATGTTTCTATATTGAACAGAAACTGCGCGAGCGGATGAATATTCCGGTATTCCACGACGATCAGCACGGCACGGCAATTATCAGCACTGCCGCCATCCTCAACGGCTTGCGCGTGGTGGAGAAAAACATCTCCGACGTGCGGATGGTGGTTTCCGGCGCGGGTGCCGCAGCAATCGCCTGTATGAACCTGCTGGTAGCGCTGGGTCTGCAAAAACATAACATCGTGGTTTGCGATTCAAAAGGCGTTATCTATCAGGGCCGTGAGCCAAACATGGCGGAAACCAAAGCCGCATATGCGGTGGTGGATGACGGCAAACGTACCCTCGATGATGTGATTGAAGGCGCGGATATTTTCCTGGGCTGTTCCGGCCCGAAAGTGCTGACCCAGGAAATGGTGAAGAAAATGGCTCGTGCGCCAATGATCCTGGCGCTGGCGAACCCGGAACCGGAAATTCTGCCGCCGCTGGCGAAAGAAGTGCGTCCGGATGCCATCATTTGCACCGGTCGTTCTGACTATCCGAACCAGGTGAACAACGTCCTGTGCTTCCCGTTCATCTTCCGTGGCGCGCTGGACGTTGGCGCAACCGCCATCAACGAAGAGATGAAACTGGCGGCGGTACGTGCGATTGCAGAACTCGCCCATGCGGAACAGAGCGAAGTGGTGGCTTCAGCGTATGGCGATCAGGATCTGAGCTTTGGTCCGGAATACATCATTCCAAAACCGTTTGATCCGCGCTTGATCGTTAAGATCGCTCCTGCGGTCGCTAAAGCCGCGATGGAGTCGGGCGTGGCGACTCGTCCGATTGCTGATTTCGACGTCTACATCGACAAGCTGACTGAGTTCGTTTACAAAACCAACCTGTTTATGAAGCCGATTTTCTCCCAGGCTCGCAAAGCGCCGAAGCGCGTTGTTCTGCCGGAAGGGGAAGAGGCGCGCGTTCTGCATGCCACTCAGGAACTGGTAACGCTGGGACTGGCGAAACCGATCCTTATCGGTCGTCCGAACGTGATCGAAATGCGCATTCAGAAACTGGGCTTGCAGATCAAAGCGGGCGTTGATTTTGAGATCGTCAATAACGAATCCGATCCGCGCTTTAAAGAGTACTGGACCGAATACTTCCAGATCATGAAGCGTCGCGGCGTCACTCAGGAACAGGCGCAGCGGGCGCTGATCAGTAACCCGACAGTGATCGGCGCGATCATGGTTCAGCGTGGGGAAGCCGATGCAATGATTTGCGGTACGGTGGGTGATTATCATGAACATTTTAGCGTGGTGAAAAATGTCTTTGGTTATCGCGATGGCGTTCACACCGCAGGTGCCATGAACGCGCTGCTGCTGCCGAGTGGTAACACCTTTATTGCCGATACATATGTTAATGATGAACCGGATGCAGAAGAGCTGGCGGAGATCACCTTGATGGCGGCAGAAACTGTCCGTCGTTTTGGTATTGAGCCGCGCGTTGCTTTGTTGTCGCACTCCAACTTTGGTTCTTCTGACTGCCCGTCGTCGAGCAAAATGCGTCAGGCGCTGGAACTGGTCAGGGAACGTGCACCAGAACTGATGATTGATGGTGAAATGCACGGCGATGCAGCGCTGGTGGAAGCGATTCGCAACGACCGTATGCCGGACAGCTCTTTGAAAGGTTCCGCCAATATTCTGGTGATGCCGAACATGGAAGCTGCCCGCATTAGTTACAACTTACTGCGTGTTTCCAGCTCGGAAGGTGTGACTGTCGGCCCGGTGCTGATGGGTGTGGCGAAACCGGTTCACGTGTTAACGCCGATCGCATCGGTGCGTCGTATCGTCAACATGGTGGCGCTGGCCGTGGTAGAAGCGCAAACCCAACCGCTGTAA |

**Table S2**. Primers used in this study.

| **Name** | **Sequence (5’-3’)** |
| --- | --- |
| SP-F | CCACGCGTAAGCTTGGATCCGCTCACGCAACTGGTCCAGAA |
| *SP-R* | CGGGAGCTCGAATTCTAGAGTGCTTAGTGCATCTAACGC |
| Pcpc-F | CGTCTAGAGGATCCCCTGTAGAGAAGAGTCCCTG |
| Pcpc-R | TTTCTCCTCTTTTGAATTAATCTCCTACTTGACTTTATGAG |
| PetE-F | GCTCTAGACAAGGATTCATAGCGGTTGCCCAATC |
| PetE-R | GCTGCCTAGGATTCTGGCGAAAGGGGGATGTG |
| TrbcL-F | CGCGTCGACCGGTGTTTGGATTGTCGGAGT |
| TrbcL-R | CCGACGCGTAAGCTTCCGGTAATTGGTAAATTGCTGTC |
| Pdc-F | CCGAGATCTCATATGTCCTACACCGTGGGCACCT |
| Pdc-R | CGCGGATCCTGCAGCTCGAGTCTAGATTACAACAATTTGTTCACGGGT |
| YqhD-F | CAAACTCGAGTCTAGATGAACAACTTTAACTTGCACACCCCCAC |
| YqhD-R | CGGGGTACCTGCAGTTAGCGGGCGGCTTCGTATATACGGC |
| slr0168Up-F | GGCATGCCGAGCGGCACCACGGGGCACCACCGC |
| slr0168Up-R | GACGCGTCGGCGCACAGCAGCGTGCGACGTGTG |
| slr0168Dw-F | CTCTAGAGTGCCACTACCTGGCGTGCCGCTACC |
| slr0168Dw-R | GGGGTACCCCGCATGACCAGCTGCCGCCCCAGC |
| slr0301Up-F | ACATGCATGCATTAACTCCCGCAGAAAGGGA |
| slr0301Up-R | CGACGCGTCGACATCATGGGTGCCCACCTCTTCA |
| slr0301Dw-F | CTAGTCTAGACGGCTTCTCCATTGGCTCCAAT |
| slr0301Dw-R | CGAGCTCGGTACCGCCCTCAACCTCTCCATTTCC |
| slr1176Up-F | ACATGCATGCCAGTTAGCCTAGGCAGTGGTTC |
| slr1176Up-R | CGACGCGTCGACCGCCCCCTAAATCCCCCAATA |
| slr1176Dw-F | CTAGTCTAGAGTCGACCATTGCCGCAACGACACTG |
| slr1176Dw-R | CGAGCTCGGTACCACCAAAGAGTTATTGGCGGCTA |
| MaeB-F | AGGAGATTAATTCAAAAGAGGAGAAAATGGATGACCAGTTAAAACAAAGTGC |
| MaeB-R | ATCCAAACACCGGTCGACTGCAGTTACAGCGGTTGGGTTTGCGCT |
| CamF | ACCGGCATGCGTTGATCGGCACGTAAGAGGT |
| CamR | CAACTGCAGTTACGCCCCGCCCTGCCACTCATC |
| PDC-qF | TGCAAATTGGCTTGAAACATC |
| PDC-qR | GGCATAGCCTTCGGCACTAAA |
| YqhD-qF | AGGTGGCTAACGGCGTAGTGG |
| YqhD-qR | CTTTCAGGGCTTTCGGACCAT |
| 16s-RTF | AGTTCTGACGGTACCTGATGA |
| 16s-RTR | GTCAAGCCTTGGTAAGGTTCT |
| MaeB-qF | CGTTGCCGCACCTTGTCTTG |
| MaeB-qR | AACGCCCTTGCCTTCCATCA |
